# Daily Dose Standardization Based on Essential and Nonessential Trace Element Presence in *Berberis baluchistanica* Ahrendt Bark, Leaf, and Root

**DOI:** 10.1155/2022/6811613

**Published:** 2022-04-25

**Authors:** Zareen Gul, Ali Akbar, Saadullah Khan Leghari, Attiq Ur Rehman Kakar, Naqeebullah Khan, Javed Muhammad, Nazir Ahmad Khan, Zia Ur Rehman, Rehana Kamal, Imran Ali

**Affiliations:** ^1^Department of Botany, University of Balochistan, Quetta, 87300 Balochistan, Pakistan; ^2^Department of Microbiology, University of Balochistan, Quetta, 87300 Balochistan, Pakistan; ^3^Department of Botany, Ghazi University Dera, Ghazi Khan, Punjab, Pakistan; ^4^Department of Chemistry, University of Balochistan, Quetta, 87300 Balochistan, Pakistan; ^5^Department of Microbiology, The University of Haripur, Pakistan; ^6^Department of Animal Nutrition, The University of Agriculture, Peshawar, Pakistan; ^7^Institute of Biochemistry, University of Balochistan, Quetta, Balochistan, Pakistan; ^8^Department of Obstetrics and Gynecology, Sandeman Provincial Hospital, Quetta, Balochistan, Pakistan

## Abstract

Medicinal plants have great importance to the consumer health, as beside beneficial compounds, plants can accumulate essential and nonessential metals from soil and surrounding environments, leading to consumer health risks. Assuming this, the present study is aimed at evaluating the elemental composition and daily dose standardization based on essential and nonessential trace element presence in of bark, leaves, and roots of *Berberis baluchistanica* Ahrendt, a common medicinal plant used as a folk medicine in the region. Atomic absorption and flame emission spectroscopy were performed to analyze the presence of essential and nonessential elements manganese (Mn), copper (Cu), lead (Pb), nickel (Ni), iron (Fe), sodium (Na), and potassium (K). Among the essential elements, K was present at high concentrations in the bark (8926.98 ± 0.32 *μ*g/g), leaves (7922.77 ± 0.42 *μ*g/g), and roots (6668.5 ± 0.96 *μ*g/g) of the plant. The estimated concentration of Na was higher in leaves (1782.56 ± 0.13 *μ*g/g), followed by roots (1089.5 ± 0.71 *μ*g/g) and bark (572.8 ± 0.62 *μ*g/g). The Fe concentration varied in the range of 394.7 ± 0.3 *μ*g/g in bark, 1298.3 ± 0.54 *μ*g/g in leaves, and 1208.9 ± 0.7 *μ*g/g in roots. The trace transition element Mn was highest in leaves (42.7 ± 0.99 *μ*g/g), followed by roots (33.5 ± 0.94 *μ*g/g) and bark (22 ± 1 *μ*g/g). The Cu concentration was low, ranging from 20.1 ± 0.63 to 22.67 ± 0.7 *μ*g/g in leaves, bark, and roots. The obtained concentration of nonessential element Pb was relatively lower than the permissible range (10 mgL–1) established by the World Health Organization. The elemental concentrations in all parts were within the set limits for provisional tolerable daily maximum intake (PTDMI) and provisional tolerable weekly intake (PTWI), and the hazard quotient index (HQ) was below 1 for all toxic metals. The micro and macroelemental distribution and the overall medicinal potential of any medicinal plant can be correlated for dose risk estimation, which will be useful in providing knowledge regarding the contraindication associated with folk medicines. In the present study, based on the elemental composition, it was calculated that the daily safe dose for *Berberis baluchistanica* is approximately 2-5 g/day of raw powder for an adult, which must not be exceeded to this safe range.

## 1. Introduction

Plants of medicinal impacts have great importance to the health of individuals and communities [1]. Medicinal plants are traditionally used for the treatment of various diseases [2]. Crude extracts of these plants comprise a mixture of different valuable bioactive components such as alkaloids, polyphenols, flavonoids, terpenoids, and minerals. These components have major antioxidant, antimicrobial, anti-inflammatory, chemo-preventative, and cytotoxic potentials. It provides safety against several diseases. Herbal medicines containing these phytochemical constituents (plant secondary metabolites) are used as alternates for synthetic and allopathic drugs and are thought to be less hazardous to humans and the environment compared to the counterpart [3]. Consumption of these medicinal plants contributes to the intake of different less important phytochemical components and other associated essential and nonessential trace elements. Along with other potential positive impacts of these plants on human health, trace elements have substantial roles in curing a variety of human diseases [4]. However, the higher concentration of these elements is also responsible for toxicity, which can even make the best drug a potent poison if the concentration of elements present in these plants exceeds the permissible specific toxicological parameters controlled by regulatory authorities. Due to their effectiveness in treating a variety of diseases, it is important to determine the concentration of these trace elements in all parts of plants along with their pharmacological properties [5].

*Berberis* is a recognized genus of the family *Berberidaceae*, containing approximately 550 species [6]. It is one of the most primitive angiosperms with great economic and medicinal value, as it contains berberine as a major phytochemical [7]. Different phytochemicals, such as glycosides, steroids, anthraquinones, saponins, alkaloids, phlobatanins, tannins, reducing sugars, flavonoids, and terpenoids, are also present in Berberis. The antioxidant, antidiabetic, anti-inflammatory, hepatoprotective, and hypotensive properties of berberine and berbamine found in Berberis plants have also been reported [8]. However, efficient investigations on the elemental concentrations of *Berberis baluchistanica* species are lacking.

*Berberis baluchistanica* Ahrendt is a wild medicinal plant locally known as Zralag in Pashto, Archin in Brahvi, and Korae in Balochi language. It is endemic to Balochistan and belongs to the family Berberidaceae. The plant is distributed in the Harboi mountain range in Kalat, Zarghun mountain in Quetta, and in Ziarat Balochistan Pakistan [9]. This medicinal plant is valued for its bark and roots and considered nontoxic and consumed in raw form, either as powder or a decoction. As it contains berberine, the plant is used for the treatment of various diseases, such as cough, fever, internal injury, eye disease, kidney stone removal, wound healing, rheumatism, and other infections of humans and livestock [10]. Previously, various secondary metabolites, such as berberisinol, berberine [11], 8-oxoberberine, oleanolic acid, palmatine [12], gallic acid, phenols, carotenoids, and vitamins [8], were isolated and found to have remarkable antioxidant, antileishmanial, antidiabetic, antibacterial, and antifungal potential [9, 10].

Traditional medicinal plants are rich in various essential and nonessential elements. These elements may be hazardous if consumed for longer periods without any restrictions. Hence, a comprehensive examination is considered significant to test the plants' essential and nonessential elements. This can be accomplished by assessing the harmful impacts of the components in light of the average upper admissible level and afterward assessing the harmfulness of elements taken from medicinal herbs. Consequently, it is necessary to quantify the essential and toxic elements along with other phytochemicals present in medicinal plants [13]. Taking into account the importance of trace elements in general well-being as well as their curative properties, medicinal plants are therefore being explored and have attracted interest regarding the effects of bioactive components, quantification of their essential and nonessential elements [14].

The present study is aimed at analyzing the elemental composition and its quantification in bark, leaves, and roots of *Berberis baluchistanica* by atomic absorption spectroscopy (AAS) and flame emission spectroscopy. Furthermore, health risks associated with nonessential elements were assessed, and the medicinal properties of all parts of the plant and their elemental distribution were correlated.

## 2. Materials and Methods

### 2.1. Plant Collection

*Berberis baluchistanica* plants were collected from Ziarat of Balochistan and were identified by taxonomist, Dr. Saadullah Khan Leghari Professor in Department of Botany University of Balochistan Quetta. The voucher specimens were prepared, and the plant was deposited in the Herbarium, Department of Botany, University of Balochistan Quetta, Pakistan.

The research work and the plant collection permission have been granted by the local and institutional authorities (no. UoB/Reg:/GSO/464 dated 01/06/2021).

### 2.2. Plant Sample Preparation

The bark, leaves, and roots of the plant were separated from each plant, washed off with distilled water to remove any kind of dust and contamination, and dried under shade for 3-4 consecutive weeks at room temperature under controlled humidity. The dried roots were separately ground into fine powder using an electrical grinder and stored in desiccators for further analysis [1].

### 2.3. Reagents and Solutions

All analytical grade standards of metals (Mn, Cu, Pb, Ni, Fe, Na, and K) were purchased from Merck. Standard solutions (0.1–20 mgL–1) of these metals were diluted in distilled water, and digestion was carried out with a mixture of HNO_3_, H_2_SO_4_, and HClO_4_ at a ratio of 5 : 2 : 1.5.

### 2.4. Instrument and Glassware

An atomic absorption spectrophotometer (AA 7000 Shimadzu) was used for the determination of heavy metals with hollow cathode lamps of different metals and a flame of air-acetylene. Na and K were detected using a flame emission spectrophotometer (Jenway PFP7). Various glassware including conical flasks, round bottom flasks, and different beakers were used. All glassware were washed, dried, soaked in an HCl bath (10% *v*/*v*) for one week, and rinsed several times with deionized water before use.

### 2.5. Sample Digestion and Determination of Trace Elements

Digestion of the powdered plant part was accomplished according to a previously reported protocol [15]. Hence, 0.50 g powder of each sample was placed in a 50 mL round bottom flask, and a 8.5 mL mixture of acids (5 mL nitric acid, 2 mL sulfuric acid, and 1.5 mL perchloric acid) was then added. After 24 h, the flasks were heated for 30 min at 60°C, further heated at 150°C for 15 min and allowed to settle with white fumes. The digestion mixture was then transferred into a 50 mL volumetric flask, and 50 mL of distilled water was poured into the bottles, followed by filtration using filter paper (Whatman No. 1). These prepared solutions after wet digestion were studied for the detection of elements using AAS and a flame photometer. Dilutions of different concentrations were prepared from analytical grade stock standards of 1000 mgL–1 for the purpose of calibration. These dilutions were prepared immediately before running the samples. Deionized water was used throughout the investigation. For quality assurance and quality control purposes, blanks were included in each batch of samples analyzed. Atomic absorption spectrophotometry was used with hollow cathode lamps of different metals as radiation sources operated at 5 mA at 393 and 279 nm wavelengths, respectively, with optimized air acetylene flames. A flame emission spectrophotometer (Jenway PFP7) was applied for the evaluation of sodium and potassium. To obtain results within the range of the flame photometer, the samples were diluted 100-fold with deionized water. The results were expressed in (*μ*g/g).

### 2.6. Hazard Quotient Assessment

Toxic metal intake causes health risks, when its consumption exceeds the set values for the upper daily intake level of a nutrient that is considered safe. For some elements, the upper daily intake level has not been established, so the health risk can be assessed by simple comparison with specific toxicological parameters controlled by regulatory authorities, i.e., the provisional maximum tolerable daily intake (PMTDI) or the provisional tolerable weekly intake (PTWI).

The health risk associated with the consumption of the toxic elements of each sampled part of the plant was evaluated by calculating the daily metal intake (DMI) and provision for weekly intake (PWI) of the metals [16]. All the calculated DMIs of metals from bark, leaves, and roots are presented in the results section.

### 2.7. Daily Metal Intake

The daily metal intake DMI (Eq. (1)) was calculated according to the equation [17, 18] with a slight modification

(1) $$\begin{array}{c} \text{DMI}=\left(C\right)\times \frac{\text{DI}}{\text{BW}}, \end{array}$$

where *C* represents the individual concentration of a toxic metal, DI represents the daily intake of metals per day, and BW is the bodyweight of an average individual assuming 70 kg.

The PWI was calculated by multiplying the daily metal intake by seven (Eq. (2)).

(2) $$\begin{array}{c} \text{PWI}=\text{DMI}\times 7, \end{array}$$

where number 7 refers to the number of days in the week.

The obtained PWI values were compared with the PTWI of the metals as recommended by the World Health Organization (WHO) and other authorities for an average adult of 70 kg body weight.

Another way to calculate the associated intake risk from medicinal plant intake is the hazard quotient (HQ), which depends on the estimated weekly metal intake (PWI) and is inversely proportional to the oral reference dose (RfD) (Eq. (3)).

(3) $$\begin{array}{c} \text{HQ}=\frac{\text{PWI}}{\text{RfD}}. \end{array}$$

RfD was used to determine the safe intake for HQ, with the following values for each element: iron 800 *μ*g/kg/day, manganese 140 *μ*g/kg/day, and nickel 15 *μ*g/kg/day [19]. For elements having no RfD values, we used the PTWI values to determine the HQ, lead 25 *μ*g/kg/week, and copper 3.5 mg/kg/week or 3500 *μ*g/kg/week.

The HQ calculus calculates the potential risk to noncarcinogenic chronic damage to human health, where HQ > 1 equals a hazard potential [20].

### 2.8. Statistical Analysis

All the experiments were conducted in triplicate and the results were expressed as the average ± standard deviations (SD). The magnitude of the means, standard curve, and standard deviations were calculated by using MS Excel 2010 Software. To identify significant elements, the obtained overall data were subjected to principal component analysis (PCA) using XLSTAT software.

## 3. Results and Discussion

**Figure 1:**
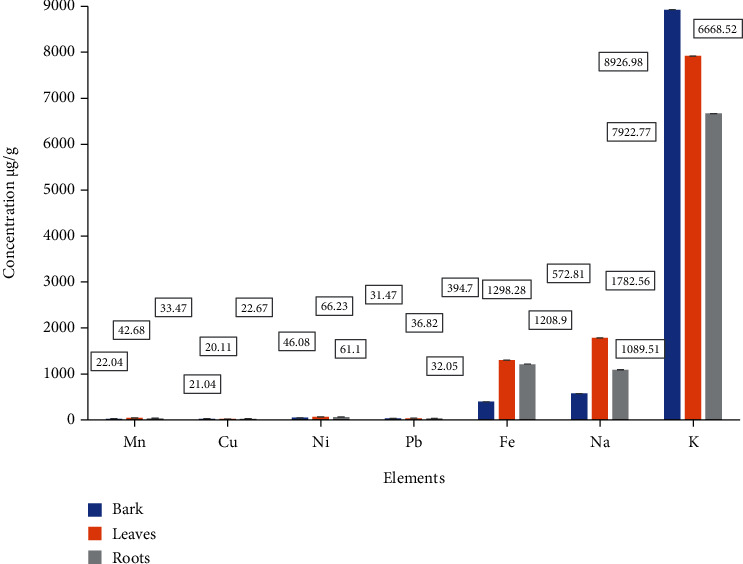
Comparative analysis of the concentrations of various metals in bark, leaves, and roots of *Berberis baluchistanica.*

The AAS is a reliable analytical technique that gives the calculated concentration of various elements. Among the essential elements, K was present at high concentrations of 8927 ± 0.3 *μ*g g − 1 in bark, 7922.8 ± 0.4 *μ*g g − 1 in leaves, and 6668.5 ± 0.96 *μ*g g − 1 in roots of the plant. The estimated concentration of Na was highest in leaves (1782.6 ± 0.13 *μ*g g − 1), followed by roots (1089.5 ± 0.7 *μ*g g − 1) and bark (572.8 ± 0.6 *μ*g g − 1). The Fe concentration varied in the range of 394.70 ± 0.3 *μ*g g − 1 in bark, 1298.3 ± 0.5 *μ*g g − 1 in leaves, and 1208.9 ± 0.7 *μ*g g − 1 in roots. The trace transition elements Mn and Cu were found in low quantities ranging from 22 ± 1 − 42.7 ± 1 *μ*g g − 1 and 20.1 ± 0.63 − 22.7 ± 0.7 *μ*g g − 1. The obtained concentrations of the nonessential elements Pb were 31.5 ± 0.98 *μ*g g − 1 in bark, 36.8 ± 1.2 *μ*g g − 1 in leaves, and 32.1 ± 1.03 *μ*g g − 1 in root samples (Figure 1 and Supplementary Table 1). All the elemental composition of the three parts was relatively lower than the permissible ranges established by the WHO.

Different parts of *Berberis baluchistanica* plants are sold in herbal medicines markets of Balochistan, because of their traditional and highly curative properties with the vernacular name Zaralg. Therefore, quality assurance of the plant has become necessary to establish the critical limits for the daily dosage uses for community. Along with the therapeutic effects, the present study is aimed at evaluating the elemental composition and daily dose standardization based on essential and nonessential trace element presence in of bark, leaves, and roots of *Berberis baluchistanica*. Among the seven detected elements, Fe, Na, K, Mn, and Cu have been classified as essential elements, while Ni and Pb are nonessential elements for the human body. All elements detected in the plant are used in the treatment of different diseases.

### 3.1. Daily Intake of Essential and Nonessential Metal at Nontoxic Levels

The DMI values for the evaluated metals in the present study are shown in Table 1. The results revealed that the DMI value for Mn in leaves (1707.21 *μ*g kg − l) was higher than that in roots (1339.80 *μ*g kg − l) and bark (881.61 *μ*g kg−1) for adults weighing 70 kg. The DMI of Cu in roots (810.32 *μ*g kg − l) was also relatively higher than that in bark (751.22 *μ*g kg − l) and leaves (718.41 *μ*g kg − l). The DMI value of Ni in bark was 16.51 *μ*g kg − l, which was lower than that in leaves (23.71 *μ*g kg − l) and roots (21.82 *μ*g kg − l). The DMI value for Pb in leaves (218.32 *μ*g kg − l) was higher than that in roots (190.24 *μ*g kg − l) and bark (186.61 *μ*g kg − l). The DMI of Fe in leaves (278202.43 *μ*g kg − l) was higher than that in roots (259050.52 *μ*g kg − l) and bark (84578.61 *μ*g kg − l). The DMI value of K in bark was 446349.02 *μ*g kg − l, which was higher than that in leaves (396138.50 *μ*g kg − l) and roots (333426.01 *μ*g kg − l). For Na the DMI value in bark was 12247.51 *μ*g kg − l, which was lower than that in leaves (38197.70 *μ*g kg − l) and roots (23346.64 *μ*g kg − l). Overall, the concentration of toxic metal daily intake was normal and below the permissible limits in all studied parts of the plant.

### 3.2. Hazard Quotient and Provision for Weekly Intake

The obtained PWI values and the HQ for evaluated metals are shown in Table 2. The results revealed that the obtained PWI values for manganese in bark (6171.12 *μ*g week−1), leaves (11950.41 *μ*g week-1), and roots were 9371.62 *μ*g week-1. The HQ values for all evaluated parts of the plant were below the toxic limit when HQ < 1. The detected (PWI) values of copper were 5259.83 *μ*g week-1 for bark, 5027.51 *μ*g week-1 for leaves, and 5667.54 *μ*g week-1 for roots with HQ < 1 for bark (0.02) and leaves (0.02) and 0.023 for roots. The highest detected (PWI) value of nickel was 165.67 *μ*g week-1 for leaves, followed by roots (152.75 *μ*g week-1) and 115.32 *μ*g week-1 for bark. The HQ values for bark were (0.02), leave (0.02), and roots (0.02). The quantified HQ values of lead were HQ < 1 for bark (0.75), leaves (0.87), and roots (0.76), and the obtained PWI values were 1306.20 *μ*g week-1 in bark (1528.31 *μ*g week-1) in leaves and 1330.12 *μ*g week-1 in roots. As iron is not considered to have any harmful health effect, except when taken at extremely large doses, the HQ values for iron in all evaluated plant parts in the present study are in safe limits. Although it is unlikely to reach the toxic amount by taking the plant in raw form, their use, along with other iron sources, must be checked not to cause chronic intoxications.

Manganese is good antioxidant and an important element for plant and animal growth. Its deficiency causes reproductive problems in mammals, and excessive amount leads to different lungs and brain diseases [21]. The obtained concentrations of manganese in bark were 22 ± 1 *μ*g g − 1, and those in leaves (42.7 ± 1 *μ*g g − 1) and roots were 33.5 ± 1 *μ*g g − 1. The permissible limit of Mn is 20 mg kg−1 for edible plants, the acceptable dietary intake of manganese is 2-5 mg day−1 [22], and the upper limit for manganese is 11 mg day−1. Based on the estimated PMTDI for Mn (140 *μ*g kg−1 bw day−1), which corresponds to 980 *μ*g kg−1 bw week−1 and 68600 *μ*g or 68.6 mg week−1 for an adult weighing 70 kg are considered to be suitable for human adults, as shown in Table 3. The results are lower than those previously reported in the literature [23, 24] and agree with the results documented by other researchers in medicinal plants [25].

Copper is an essential element necessary for plant growth and development. It plays an important role in metabolic processes and regulates alkaloid accumulation in medicinal plants. It is used as an ailment for wound inflammation and chest and arthritis [29]. However, severe exposure to high levels of copper is associated with inflammation of brain tissues, hair loss, allergies, depression, and liver and renal damage [30]. According to Mishra et al. ^4^, the Cu concentration varies between 2 and 20 mgL–1, the permissible limit of copper in plants is 10 mg kg−1, and the dietary intake for an average human body is 2–3 mg day−1. The FAO/WHO has established a PTWI of 3.5 mg kg−1 body weight 3.5 mg kg−1 week−1 for an adult weighing 70 kg which corresponds to 245 mg week−1 [31]. The concentrations of copper in bark, leaves, and roots were 21 ± 1 *μ*g g − 1, 20.1 ± 0.63 *μ*g g − 1, and 22.7 ± 0.7 *μ*g g − 1, respectively, and the order of concentration was roots > bark > leaves. The obtained results are below the lower limit, as shown in Table 3. The copper concentrations in all samples are recommended for human adults. Comparing our results, Cu was found to be lower in concentration than previously reported [24]. The copper concentrations in all parts (Supplementary Table 1) were below the PTWI and HQ < 1. According to Dos Santos et al. ^20^, copper requirements for an adult are 900 *μ*g day−1; therefore, the medicinal plants studied cannot be considered a source of this element.

Nickel is an important element that controls several metabolic processes in plants. It is required for the insulin production. Its deficiency leads to cardiac function, loss of body weight, and liver problems. However, nickel is toxic at higher concentrations and shows carcinogenic side effects when consumed in high quantities [32]. High concentrations of nickel cause severe chlorosis and necrosis and anatomical changes in plants. Nickel has been reported to cause allergic problems in humans and is adversely harmful to the lungs and nasal cavities [15]. The estimated PTWI [31] for Ni is 15 *μ*g kg−1 day−1 for humans, which corresponds to 105 *μ*g kg−1 bw week−1 and 7350 *μ*g week−1 for adults weighing 70 kg. The obtained concentrations of nickel (46.08 ± 0.28 *μ*g g − 1 in bark, 66.23 ± 0.33 *μ*g g − 1 in leaves, and 61.1 ± 1 *μ*g g − 1 in roots) and the PWI for bark (115.32), leaves (165.67), and roots (152.75 *μ*g week−1) were below the provisional tolerable weekly intake and HQ < 1, as presented in Table 2.

Lead is a nonessential heavy metal. The use of Pb makes it unique regarding environmental toxicity, which is why it is very important to study heavy metals such as Pb [33]. Its deposition in the soft tissues, renal, immune, and nervous systems of the body affects its functions. The safe limit of lead for human use is 1.5 mgL–1, while in medicinal plants, the permissible limit is 10 mgL–1 [25]. It was noted that the highest Pb concentration was determined in leaves (36.8 ± 1.2 *μ*g g − 1), followed by 32.1 ± 1.03 *μ*g g − 1 in roots and 31.5 ± 1 *μ*g g − 1 in bark of the plant. The presence of a relatively higher concentration of Pb in leaves may be due to airborne lead. According to the FAO/WHO, the PTWI is 25 *μ*g lead kg−1 or 0.025 mg kg−1 body weight for humans, which is 1.5 mg week−1 [31]. There is no upper limit set for lead yet [20]; however, the obtained Pb concentrations detected in all samples were below the permissible limits set by the WHO, and HQ < 1 shows noncarcinogenic chronic damage to human health. The obtained concentration of this element was relatively lower than those found in the literature [34]. The daily dietary intake of lead for a person weighing 70 kg was approximately 0.42 mg (Table 3), and all parts had concentrations below the safe limits.

Iron is one of the most important elements known to produce red blood cells in the human body. Fe is an essential trace element (micronutrient) for living organisms. Its deficiency causes anemia, and its high amount damages tissues in the human body. Generally, iron is not considered to have harmful health effects, except when taken at extremely large doses. The acceptable limit of iron in plants is 20 mg kg−1, while it is 10 to 28 mg day−1 for human consumption [25]. The JECFA established a provisional maximum tolerable daily intake (PMTDI) of 0.8 mg/kg of body weight. The prescribed PMTDI value corresponds to 5.6 mg kg−1 bw week−1, as 392 mg week−1 for an adult weighing 70 kg. The results obtained in the present work were 394.7 ± 0.29 *μ*g g − 1 in bark, 1298.3 ± 0.54 *μ*g g − 1 in leaves, and 1208.9 ± 0.71 *μ*g g − 1 in roots. The metal concentration was higher in leaves than in roots and bark. The obtained results are in agreement with previously reported data [35, 36]. The Fe concentration suggests the possible use of this medicinal plant to compensate for iron deficiency.

Sodium is an essential element that regulates fluid balance and proper functioning of muscles and nerves. Its deficiency causes mood swing, dehydration, and hair loss. According to the results, the highest concentration (1782.6 ± 0.1 *μ*g g − 1) of Na was present in leaves, followed by 1089.5 ± 0.7 *μ*g g − 1 in roots, and a lower concentration (572.8 ± 0.6 *μ*g g − 1) was present in bark. The concentration of Na in *Berberis baluchistanica* was found to be in the order of leaves > roots > bark. The adequate intake range is 1500-2300 mg day−1 for an adult with 70 kg body weight [37]. The DMI value of Na in bark was 12247.51 *μ*g kg − l, which was lower than that in leaves (38197.70 *μ*g kg − l) and roots (23346.64 *μ*g kg − l). The average concentration of Na present in plants is reasonably high, making this plant medicinally important. However, the obtained results showed that Na was low enough, indicating a satisfactory concentration of this element, as reported in other medicinal plants [15, 23, 38, 39].

Potassium is one of the well-known essential elements that regulate the responses of the immune system and hormones. It acts as an activator of some enzymes and works in the secretion of insulin and is involved in lowering blood pressure and kidney problems. The average concentration of K was 8927 ± 0.3 *μ*g g − 1 in bark, 7922.8 ± 0.4 *μ*g g − 1 in leaves, and 6668.5 ± 1 *μ*g g − 1 in roots. The order of concentration was bark > leaves > roots, and the adequate intake range is 3500–4700 mg day−1 for an adult with 70 kg body weight. The DMI value of K in bark was 446349.02 *μ*g kg − l, which was higher than that in leaves (396138.50 *μ*g kg − l) and roots (333426.01 *μ*g kg − l). Recently, Uddin et al. [9] reported that the K concentration (0.74%) from *Berberis baluchistanica* stems by energy dispersion spectroscopy was lower than the present result. The obtained results were relatively higher than those found in the literature [15, 40]. However, the results were low enough compared with those previously reported [38, 39]. There is neither any upper tolerable level set for potassium ingestion nor any reference values for weekly or monthly intake [20]. In healthy populations, excess potassium is excreted through the kidneys [41]. Therefore, the potassium amount in medicinal plants is unlikely to cause any side effects.

### 3.3. Principal Component Analysis

The PCA was used to determine the multidimensional overview of a multivariate data, which changes a bunch of factors, related to one another, in other uncorrelated, free, and symmetrical, principal components (PC) that are the outcome of direct blends of the first factors. The purpose is to regulate the components that clarify however much as could reasonably be expected of the information complete variety, with the smallest probable number of extents. They are designed in descending order of significance, i.e., the PC1 depicts the variation in the data, and the second, since it is symmetrical, clarifies a significant part of the remaining variation, to such an extent that the last component will be the one that gives minimal contribution to the clarification of the complete variance of the actual data [42].

**Figure 2:**
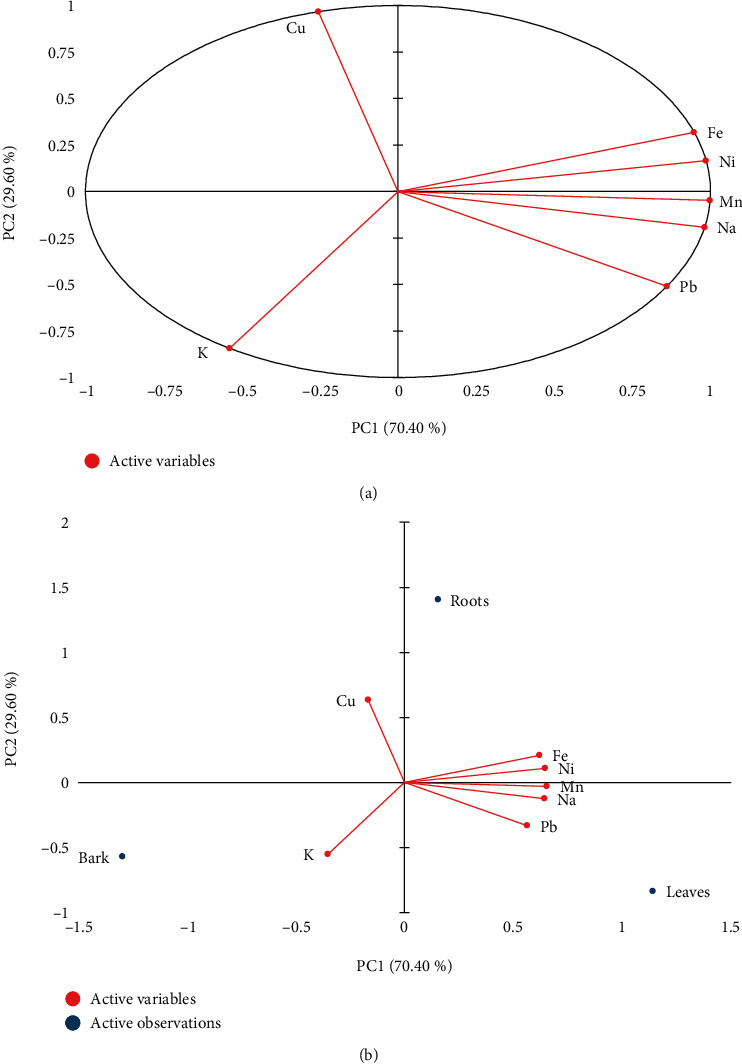
(a, b) Biplot diagrams of the original variables ordering and the scores in the first two principal components 1 and 2.

The quantified elements (Mn, Cu, Pb, Ni, Fe, Na, and K) were used as variables in the PCA. By extracting the PCs with significant eigenvalues greater than 1, two principal components were produced. Analyzing the eigenvalues, it is observed that it is possible to consider only two principal components, because from this value, there are no major changes in the accumulated variance. PC1 with highest eigenvalue of 4.93 was explaining the maximum variability in the obtained set of data followed by PC2 2.07. These principal components contributed for 100% of the total variance. The first and second components showed a variance of 70.4% and 29.6%, respectively. PCA biplot in Figure 2 shows the variability between the elements, independent variables, and the investigated dependent variables. PCA variable loadings showed higher percentage contributions (Supplementary Table 2) and a positive correlation on PC1 with Mn, Pb, Ni, Fe, and Na, and negative ones with Cu and K. PC2 was positively correlated with Cu and negative ones with K which the eigenvectors gained the negative signal in PC1. The correlation between the original variables and PC1 is also negative, indicating inverse quantities acting in contrast, that is, the higher the value of Mn, Ni, Na, Fe, and Pb, the greater the contributions of these variables in the PC1, and consequently, the lower the values of the principal component from the linear combination of the original variables. From PCA biplot case score, it is evident that leaves have the highest concentration of Mn, Na, and Pb. Roots showed higher amount of Fe, Ni, and Mn. From the PC2 scores, it is evident that bark has the highest concentration of K, respectively. The overall PCA results showed that relatively higher concentration of Mn, Pb, Ni, Fe, and Na distinguished leaves from bark and roots. PCA, further, revealed that the elemental composition of leaves is a potential outlier due to its accumulation affinity for these elements.

## 4. Conclusion

The concentrations of seven essential and nonessential elements were estimated in the bark, leaves, and roots of the plants, among which the concentrations of potassium and sodium were found to be higher in all three parts. The concentrations of toxic and heavy metals detected were within the permissible values established for the PMTDI and PTWI limits. All studied parts had elemental concentrations below the upper limit, with HQ values below one. Based on the presence of different elements, it has been calculated that a 2-5 g day−1 dose of raw *Berberis baluchistanica powder* is a safe dose for an adult to be used. Therefore, the use of this plant must not be exceeded to this safe range. The plant is nontoxic to use, and the medicinal qualities of the plant can be linked to their rich amounts of essential elements. The results obtained in the present study will support the safe use of all parts of the plant in traditional medicines for curing various diseases.

## Figures and Tables

**Table 1:** Daily metal intake (*μ*g day-1) for an adult weighing 70 kg from bark, leaves, and roots of *Berberis baluchistanica.*

Heavy metals	DMI from bark (*μ*g day-1 person-1)	DMI from leaves (*μ*g day-1 person-1)	DMI from roots (*μ*g day-1 person-1)
Mn	881.61	1707.21	1339.80
Cu	751.22	718.41	810.32
Ni	16.51	23.71	21.82
Pb	186.61	218.32	190.24
Fe	84578.61	278202.43	259050.52
K	446349.02	396138.50	333426.01
Na	12274.51	38197.70	23346.64

DMI: daily metal intake.

**Table 2:** Results of PWI (*μ*g week-1) and health risk assessments expressed through (HQ).

Heavy metals	PWI bark (*μ*g week-1)	HQ	PWI leaves (*μ*g week-1)	HQ	PWI roots (*μ*g week-1)	HQ
Mn	6171.12	0.09	11950.41	0.17	9371.62	0.14
Cu	5259.83	0.02	5027.51	0.02	5667.54	0.02
Ni	115.32	0.02	165.67	0.02	152.75	0.02
Pb	1306.20	0.74	1528.31	0.87	1330.12	0.76
Fe	592050.31	1.51	1947414.43	4.96	1813350.34	4.63

PWI: provision for weekly intake; HQ: hazard quotient.

**Table 3:** ADDIs (mg day−1) by a person weighing 70 kg [26, 27] and US RDA (mg day−1) [28].

Element	ADDIs (mg day−1)	US RDA (mg day−1)
Fe	15 (10–28)	10–18
Mn	2.8 (2–5)	1.0–5.0
Cu	2.5 (2–3)	1.0–3.0
Ni	0.025	0.13–0.4
Pb	0.415	∗∗∗
Na	∗∗∗	1500-2300
K	3500–4700	3500–4700

ADDIs: average daily dietary intake; US RDA: recommended daily dietary allowance; ∗∗∗: data not available.

## Data Availability

The data will be available on reasonable request.

## Additional Points

*Statement regarding Plant Collection*. The plant collection and use were in accordance with all the relevant guidelines.

## Supplementary Materials

Supplementary Materials Supplementary Table 1: concentration (*μ*g/g) of various elements in different parts of *Berberis baluchistanica.* Supplementary Table 2: standardized eigenvectors, correlation matrix, and the percentage contribution of each original variable in the principal components.
