# Supplementary material for: Daily Dose Standardization Based on Essential and Nonessential Trace Element Presence in Berberis baluchistanica Ahrendt Bark, Leaf, and Root
Source: Biomed Res Int. 2022 Apr 25;2022:6811613. doi: 10.1155/2022/6811613 (PMC9060997; doi:10.1155/2022/6811613)
Supplement: Supplementary Materials — Supplementary Table 1: concentration (μg/g) of various elements in different parts of Berberis baluchistanica. Supplementary Table 2: standardized eigenvectors, correlation matrix, and the percentage contribution of each original variable in the principal components. [file 6811613.f1.docx]

**Supplementary tables**

**Supplementary Table 1.** Concentration (µg/g) of various elements in different parts of *Berberis baluchistanica*

| **Plant parts** | **Mn** | **Cu** | **Ni** | **Pb** | **Fe** | **Na** | **K** |
| --- | --- | --- | --- | --- | --- | --- | --- |
| **Bark** | 22±1 | 21±1 | 46.1±0.3 | 31.5±1 | 394.7±0.3 | 572.8±0.6 | 8926.1±0.3 |
| **Leaves** | 42.7±0.99 | 20.1±0.6 | 66.2±0.33 | 36.8±1.2 | 1298.3±0.5 | 1782.6±0.1 | 7922.8±0.4 |
| **Roots** | 33.5±0.94 | 22.7±0.7 | 61.1±0.97 | 32.1±1 | 1208.9±0.7 | 1089.5±0.7 | 6668.5±1 |

**Note**: Results are expressed as the mean ± SD, Mn= Manganese, Cu= Copper, Pb= Lead, Ni= Nickel, Fe= Iron, Na= Sodium, K= Potassium

**Supplementary Table 2.** Standardized eigenvectors, correlation matrix and the percentage contribution of each original variable in the principal components

| **Original variables** | **PC1** | | | **PC2** | | |
| --- | --- | --- | --- | --- | --- | --- |
|  | E1 | R | Cont. (%) | E2 | R | Cont. (%) |
| **Mn** | 0.45 | 0.1 | **20.25** | -0.03 | -0.05 | 0.11 |
| **Cu** | -0.12 | -0.26 | 1.32 | 0.67 | 0.97 | **45.12** |
| **Ni** | 0.44 | 0.99 | **19.73** | 0.12 | 0.17 | 1.33 |
| **Pb** | 0.39 | 0.86 | **15.04** | -0.35 | -0.51 | 12.49 |
| **Fe** | 0.43 | 0.95 | **18.23** | 0.22 | 0.32 | 4.91 |
| **Na** | 0.44 | 0.98 | **19.55** | -0.13 | -0.19 | 1.778 |
| **K** | -0.24 | -0.54 | 5.89 | -0.59 | -0.84 | **34.26** |

**Note**: E1. E2 = eigenvectors; Cont. (%) = percentage contributions of the original variables; R = linear correlations between the original variables and the principal components 1 and 2; In bold= variables with the most significant contributions in the components.
